# Dual-energy computed tomography with new virtual monoenergetic image reconstruction enhances prostate lesion image quality and improves the diagnostic efficacy for prostate cancer

**DOI:** 10.1186/s12880-024-01393-3

**Published:** 2024-08-12

**Authors:** Nina Fan, Xiaofeng Chen, Yulin Li, Zhiqiang Zhu, Xiangguang Chen, Zhiqi Yang, Jiada Yang

**Affiliations:** Department of Radiology, Meizhou People’s Hospital, Meizhou, 514000 Guangdong China

**Keywords:** Prostate cancer, Dual-energy computed tomography, Virtual monoenergetic images in arterial phase, Diagnostic performance

## Abstract

**Background:**

Prostate cancer is one of the most common malignant tumors in middle-aged and elderly men and carries significant prognostic implications, and recent studies suggest that dual-energy computed tomography (DECT) utilizing new virtual monoenergetic images can enhance cancer detection rates. This study aimed to assess the impact of virtual monoenergetic images reconstructed from DECT arterial phase scans on the image quality of prostate lesions and their diagnostic performance for prostate cancer.

**Methods:**

We conducted a retrospective analysis of 83 patients with prostate cancer or prostatic hyperplasia who underwent DECT scans at Meizhou People’s Hospital between July 2019 and December 2023. The variables analyzed included age, tumor diameter and serum prostate-specific antigen (PSA) levels, among others. We also compared CT values, signal-to-noise ratio (SNR), subjective image quality ratings, and contrast-to-noise ratio (CNR) between virtual monoenergetic images (40–100 keV) and conventional linear blending images. Receiver operating characteristic (ROC) curve analyses were performed to evaluate the diagnostic efficacy of virtual monoenergetic images (40 keV and 50 keV) compared to conventional images.

**Results:**

Virtual monoenergetic images at 40 keV showed significantly higher CT values (168.19 ± 57.14) compared to conventional linear blending images (66.66 ± 15.5) for prostate cancer (*P* < 0.001). The 50 keV images also demonstrated elevated CT values (121.73 ± 39.21) compared to conventional images (*P* < 0.001). CNR values for the 40 keV (3.81 ± 2.13) and 50 keV (2.95 ± 1.50) groups were significantly higher than the conventional blending group (*P* < 0.001). Subjective evaluations indicated markedly better image quality scores for 40 keV (median score of 5) and 50 keV (median score of 5) images compared to conventional images (*P* < 0.05). ROC curve analysis revealed superior diagnostic accuracy for 40 keV (AUC: 0.910) and 50 keV (AUC: 0.910) images based on CT values compared to conventional images (AUC: 0.849).

**Conclusions:**

Virtual monoenergetic images reconstructed at 40 keV and 50 keV from DECT arterial phase scans substantially enhance the image quality of prostate lesions and improve diagnostic efficacy for prostate cancer.

## Study highlights

1. Dual-energy computed tomography (DECT) with virtual monoenergetic images significantly improves the image quality of prostate lesions.
2. Virtual monoenergetic images at 40 keV and 50 keV provide higher diagnostic accuracy for prostate cancer than conventional CT images.
3. The study shows superior contrast-to-noise ratio (CNR) values in low-energy virtual monoenergetic images compared to conventional images.
4. Subjective evaluations indicate that radiologists find low-energy virtual monoenergetic images more useful for prostate cancer diagnosis.
5. DECT with virtual monoenergetic imaging could reduce the need for invasive diagnostic procedures in prostate cancer detection.

## Introduction

Prostate cancer (PCa) ranks among the most prevalent cancers in middle-aged and elderly men and is the fifth leading cause of cancer-related deaths in males [1]. Its incidence has been increasing annually due to extended life expectancy, making accurate diagnosis and grading essential for timely treatment planning and prognostic assessment [2]. Despite being a primary diagnostic tool, prostate biopsy’s invasive nature can lead to complications such as hematuria and infection [3]. Various imaging modalities are employed in PCa diagnosis, with each modality having inherent limitations [4]. Magnetic resonance imaging (MRI), while highly sensitive, is hampered by its high cost, lengthy procedure times, patient discomfort, and others [5]. Ultrasound, characterized by low spatial resolution and susceptibility to intestinal gas interference, relies heavily on operator experience, lacks specificity, and is inadequate for tumor staging [6, 7]. Similarly, computed tomography (CT) imaging may not accurately distinguish between tumor tissue, normal tissue, and prostatic hyperplasia tissue, particularly in terms of contrast [8]. Thus, there is an urgent clinical need to develop less invasive yet highly effective diagnostic methods for PCa.

In this context, advancements in medical imaging techniques, such as the ConvUNeXt model for efficient medical image segmentation, highlight the ongoing efforts to enhance diagnostic accuracy while minimizing computational resources and parameters [9]. Ansari et al. (2022) introduced Res-PAC-UNet, a lightweight neural network for liver CT segmentation, achieving a Dice coefficient of 0.950 ± 0.019 with low computational burden, improving cancer imaging workflows [10]. Similarly, Jafari et al. (2020) proposed DRU-net, combining ResNet and DenseNet advantages for efficient medical image segmentation, achieving high accuracy with reduced parameters [11]. Ansari et al. (2023) developed Dense-PSP-UNet for real-time liver ultrasound segmentation, ensuring high Dice coefficient and real-time performance, important for immediate diagnostic applications [12].

Further, Xie et al. (2021) introduced CoTr, a framework that bridges CNN and Transformer for 3D medical image segmentation, addressing the limitations of CNNs in modeling long-range dependencies. This hybrid approach significantly improved segmentation performance for multi-organ tasks, which is pertinent to enhancing PCa imaging [13]. Ansari et al. (2021) reviewed the clinical utility of liver segmentation methods in surgical and radiological interventions for HCC, emphasizing the importance of accurate segmentation in optimizing diagnosis and treatment outcomes. This underscores the relevance of precise imaging techniques in clinical decision-making [14]. Akhtar et al. (2021) assessed the impact of CAD systems in hepatic resection, finding that automatic CAD adoption correlated with quicker tumor relapse compared to non-adoption. This highlights the potential pitfalls and considerations in adopting automated imaging systems for cancer diagnosis [15]. Moreover, Rai et al. (2023) systematically reviewed fusion imaging’s efficacy for immediate post-ablation assessment of malignant liver neoplasms. They found that fusion imaging could accurately determine short-term post-ablation outcomes, indicating its potential for immediate diagnostic assessments [16].

Despite these advancements, the current literature lacks comprehensive studies focusing on the application and effectiveness of these advanced imaging techniques in PCa diagnosis, underscoring the need for further research to validate and potentially integrate them into clinical practice for PCa. Another modality, namely dual-energy CT (DECT), has been shown to enhance tissue characterization by utilizing differential X-ray attenuation at different energy levels, thereby improving tumor detection rates and diagnostic accuracy [17]. Recently, the development of virtual monoenergetic image reconstruction has emerged as a critical advancement in DECT, particularly in tumor imaging [18]. Numerous studies have demonstrated that DECT significantly enhances soft tissue resolution and lesion conspicuity compared to conventional CT. For instance, combining low-energy virtual monoenergetic images with iodine mapping has notably improved the detection of pancreatic ductal adenocarcinoma [19]. However, there remains a gap in literature regarding the application and effectiveness of DECT with virtual monoenergetic images for diagnosing PCa.

Herein, we designed this present study to assess the effect of virtual monoenergetic images reconstructed using DECT during the arterial phase on prostate lesion image quality and diagnostic performance. By comparing these images at different energy levels (40–100 keV) to conventional linear blending images and analyzing their diagnostic efficacy, we aimed to enhance PCa detection and diagnosis, which could potentially reduce the need for invasive procedures and lead to better patient outcomes.

## Materials and methods

### Study population and ethical approval

**Fig. 1:**
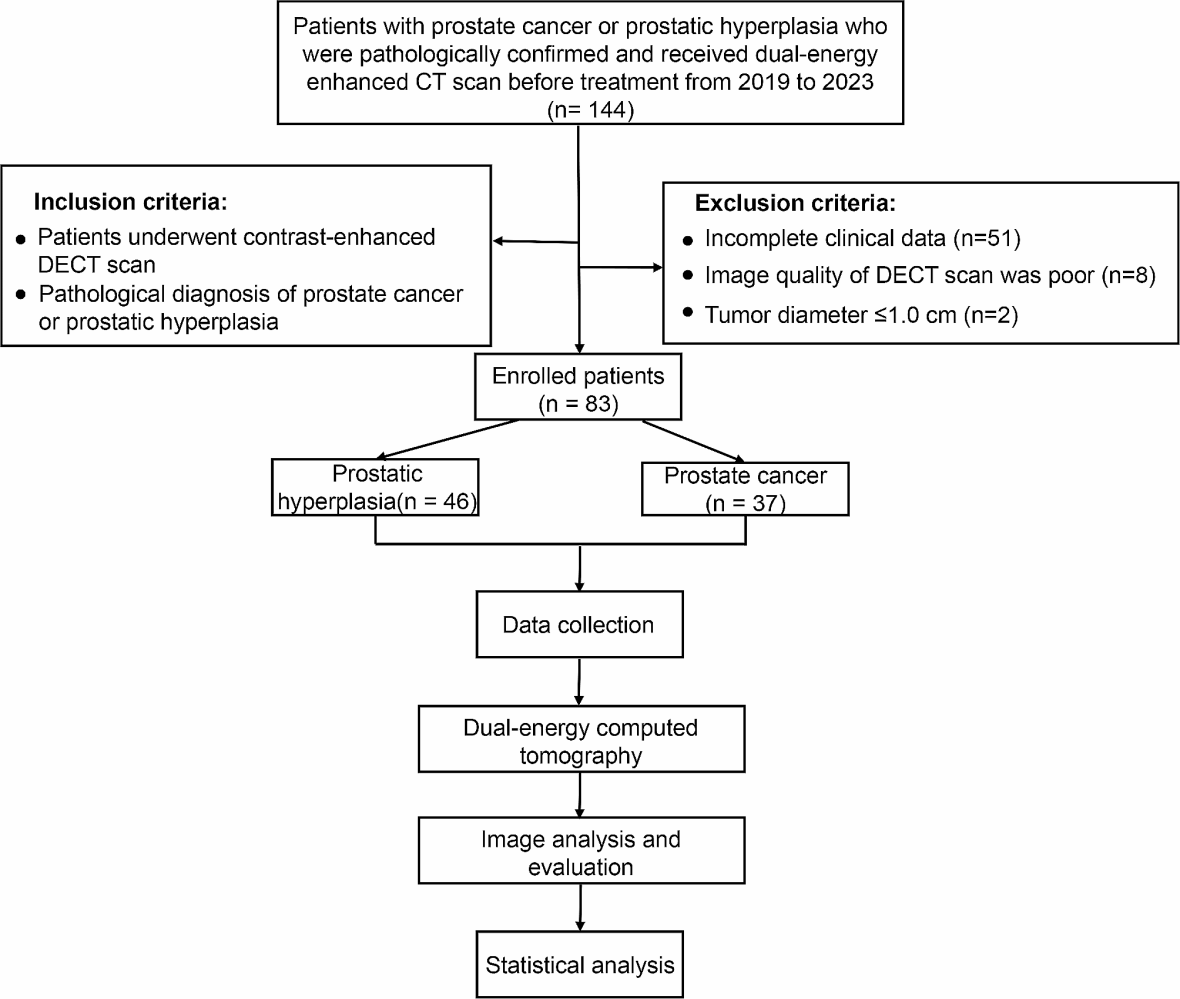
Flowchart illustrating the patient screening process for inclusion and analytic flow in the study

This study received approval from the Ethics Committee of Meizhou People’s Hospital, and informed consent was obtained from all patients or their guardians. The research adhered to the ethical standards outlined by the Committee on the Use of Human Subjects and the Declaration of Helsinki (1975). A retrospective analysis was conducted on 83 patients diagnosed with PCa or prostatic hyperplasia who underwent DECT scans at Meizhou People’s Hospital between July 2019 and December 2023. The study inclusion criteria included patients diagnosed with PCa confirmed by pathological results, those clinically diagnosed with prostatic hyperplasia based on symptoms and confirmed by ultrasound and MRI showing no suspected cancerous lesions, and individuals who underwent contrast-enhanced DECT scans. Exclusion criteria involved cases with incomplete clinical or DECT data (*n* = 51), poor DECT image quality (*n* = 8) [20], and tumors measuring less than 1.0 cm in minimal length (*n* = 2) due to the resolution limitations of DECT, which can impact the accuracy and reliability of imaging results. The patient selection process is shown in Fig. 1.

### Prostate-specific antigen detection

All patients fasted for 8–12 h before providing a fasting venous blood sample (5 mL) the next morning. Blood samples were centrifuged at 4000 r/min at 4 °C for 10 min within 2 h of collection, and the supernatant was stored at -20 °C or immediately analyzed. Total PSA (TPSA) and serum-free PSA (FPSA) concentrations were measured using electrochemiluminescence immunoassay.

### Data collection

General information on PCa patients, including age, body mass index (BMI), Gleason scores, and T staging, was collected through case investigation and follow-up forms. The reasons for patient visits (e.g., dysuria, hematuria) were also recorded. Data integrity and authenticity were verified by a second researcher after initial data collection.

### DECT scanning protocol

All patients were scanned using a third-generation dual-source Force CT scanner (Somatom Force; Siemens Healthcare, Forchheim, Germany). The scan range extended from the lower abdominal aorta to the pelvic floor. Scan parameters included: tube voltages of 100 kV and 150 snkV, automatic tube current modulation (Care Dose 4D, Siemens Healthcare), rotation speed of 0.5 s/r, pitch of 0.9–1.1, slice thickness of 5 mm with a 0.6 mm interval, and reconstructed image thickness of 1.0 mm. Iopamidol (370 mg/ml) was administered via a high-pressure syringe into the median cubital vein at a rate of 3.5 mL/s, with dosage based on body weight (1.5 ml/kg). The arterial phase scan began 5 s after reaching 100 HU in the lower abdominal aorta, followed by a venous phase scan starting 26 s later [21].

### Image analysis and evaluation

Following scanning, the conventional linear blending images of the arterial phase (fusion coefficient: 0.5) and original data from the arterial phase (100 kV and 150 snkV) were transferred to the Siemens syngo.via client workstation. The Mono-plus subroutine within the dual-energy program was employed to reconstruct data at 10 keV intervals ranging from 40 to 100 keV, yielding seven groups of monoenergetic reconstructed images (40, 50, 60, 70, 80, 90, and 100 keV). The analytical flow of this study is shown in Fig. 1.

#### Objective evaluation of the image quality

Two radiologists conducted independent and double-blind measurements, averaging their findings. Measurements included CT values of PCa foci, prostatic hyperplasia tissues, and muscles at the same layer, as well as the standard deviation (SD) of subcutaneous fat in the buttocks, defining image noise. Subsequently, signal-to-noise ratio (SNR) and contrast-to-noise ratio (CNR) were calculated using the following formulas: SNR = (CT value of cancer focus / SD) and (CT value of hyperplastic tissue / SD), CNR = [(CT value of cancer focus - CT value of muscle at the same layer) / SD] and [(CT value of hyperplastic tissue - CT value of muscle at the same layer) / SD].

#### Subjective evaluation of the image quality

Two radiologists independently and double-blindly evaluated each dataset’s image quality. In cases of discordance, consensus was reached through consultation. Image quality was scored on a 5-point scale [22], where 5 points indicated excellent, 4 points good, 3 points medium with acceptable lesion identification upon careful observation, 2 points poor contrast hindering lesion identification, and 1 point indicated insufficient diagnostic information with unidentified lesions.

### Statistical analysis

The SPSS v26.0 software was used for statistical analysis. Normally distributed measurement data are presented as mean ± standard deviation (SD). Overall comparisons were conducted using one-way analysis of variance (ANOVA) and pairwise comparisons using independent sample t-tests. Non-normally distributed data are expressed as median (interquartile range, IQR), compared overall using the Kruskal-Wallis H test, and pairwise using the Mann-Whitney U test. The diagnostic efficacy of spectral CT quantitative parameters for PCa was assessed using ROC curve analysis, with significance set at *P* < 0.05.

## Results

### Baseline data

Table 1 presents the general characteristics of all patients, comparing those diagnosed with prostatic hyperplasia (*n* = 46) and PCa (*n* = 37). Among the 37 cases of pathologically confirmed PCa,36 (97.3%) were classified as clinically significant PCa (csPCA) based on a Gleason score ≥ 7 or evidence of extraprostatic extension. The remaining 1 case (2.7%) was categorized as non-csPCA, characterized by lower Gleason scores without extraprostatic involvement. There were no significant differences in age and BMI between the two groups. However, substantial differences were noted in both free prostate-specific antigen (FPSA) and total prostate-specific antigen (TPSA) levels. Median FPSA levels were 1.30 (0.61–2.28) ng/ml for the prostatic hyperplasia group and 20.60 (3.93–30.00) ng/ml for the PCa group (*p* < 0.001). Median TPSA levels were 5.36 (2.29–10.13) ng/ml and 99.98 (28.48–386.70) ng/ml, respectively (*p* < 0.001). These biomarkers could be promising for distinguishing between prostatic hyperplasia and PCa, reflecting disease severity and potentially guiding treatment decisions.

**Table 1:** General information of patients

Eigenvalues	Prostatic hyperplasia (*n* = 46)	Prostate cancer (*n* = 37)	T/χ^2^	*P*	
Age (year)	75.46 ± 9.85	76.189 ± 8.72	0.354	0.724	
BMI (kg/m^2^)	23.22 ± 4.16	23.08 ± 3.12	-0.160	0.873	
FPSA (ng/ml)	1.30 (0.61–2.28)	20.60 (3.93–30.00)	-5.370	< 0.001	
TPSA (ng/ml)	5.36 (2.29–10.13)	99.98 (28.48–386.70)	-5.570	< 0.001	
Reasons for the visit, n (%)			4.506	0.105	
Dysuria	36 (78.26%)	27 (72.97%)			
Hematuria	2 (4.35%)	7 (18.9%)			
Others	8 (17.39%)	3 (8.11%)			
Gleason Score, n (%)	-		-	-	
6	-	1(2.7%)			
7	-	2(5.4%)			
8	-	14(37.84%)			
9	-	13(35.14%)			
10	-	7(18.92%)			
T staging, n (%)			-	-	
T1	-	0 (0.0%)			
T2	-	2 (5.4%)			
T3	-	9 (24.3%)			
T4	-	26 (70.3%)			

Note: Data were expressed as mean ± SD or median (interquartile range, IQR). BMI, body mass index; FPSA, free prostate-specific antigen; TPSA, total prostate-specific antigen

### Objective evaluation of image quality

Compared with the conventional linear blending group, the average CT attenuation values of the arterial phase in the 40–60 keV energy range were significantly elevated (*P* < 0.05), while they decreased at 100 keV. However, there were no significant differences between the 60 keV, 70 keV, and 80 keV groups compared to the conventional linear blending group (*P* > 0.05) (Table 2).

**Table 2:** Comparison of objective evaluation parameters between monoenergetic reconstruction and conventional linear blending images (CT value)

Groups	Prostatic hyperplasia (*n* = 46)	Prostate cancer (*n* = 37)	t	*P*
40 keV	93.93 ± 24.16*	168.19 ± 57.14*	7.981	< 0.001
50 keV	73.21 ± 14.92*	121.73 ± 39.21*	7.734	< 0.001
60 keV	60.08 ± 10.78*	93.91 ± 25.9*	8.047	< 0.001
70 keV	53.10 ± 7.60	76.73 ± 18.61	7.847	< 0.001
80 keV	48.98 ± 5.77	65.32 ± 13.88	7.251	< 0.001
90 keV	45.77 ± 5.07	56.76 ± 11.02*	6.022	< 0.001
100 keV	43.74 ± 4.33*	52.99 ± 8.58*	6.383	0.001
Conventional linear blending	50.70 ± 6.65	66.66 ± 15.5	6.297	< 0.001

Note: Data were expressed as mean ± standard deviation (SD). *P < 0.05 indicates a significant difference when compared to the Conventional linear blending group

Additionally, the CT values of prostatic hyperplasia in the arterial phase were significantly lower than those of PCa across various monoenergetic reconstruction energy levels (40–100 keV) and conventional linear blending groups (*P* < 0.001). Furthermore, CT values of both prostatic hyperplasia and PCa tissues exhibited a decreasing trend with increasing energy levels (40 keV, 50 keV, 60 keV, 70 keV, 80 keV, 90 keV, 100 keV) (Table 2).

Moreover, SNR and CNR values were analyzed for each group (Table 3). Significant differences were observed in SNR and CNR values between patients with prostatic hyperplasia and those with PCa across different single-energy reconstruction keV levels (40–100 keV) and conventional linear blending images (*P* < 0.001). Specifically, SNR values at each energy level did not differ significantly from those of the conventional linear blending group (*P* > 0.05) (Table 3). Compared with the conventional linear blending group, CNR values in the 40–70 keV groups for patients with prostatic hyperplasia or PCa were markedly increased (*P* < 0.05), while those in the 100 keV groups were significantly decreased (*P* < 0.05) (Table 3). Overall, these results underscore the optimal energy range for effective prostate imaging using DECT.

**Table 3:** Comparison of the signal-to-noise ratio (SNR) and contrast-to-noise ratio (CNR) values in various monoenergetic reconstruction keV levels (40–100 keV) and conventional linear blending images

Groups		Prostatic hyperplasia (*n* = 46)	Prostate cancer (*n* = 37)	t	*P*
SNR	40 keV	3.74 ± 1.31	6.17 ± 2.10	6.429	< 0.001
	50 keV	3.80 ± 1.20	6.08 ± 2.02	6.369	< 0.001
	60 keV	4.01 ± 1.15	6.18 ± 1.88	6.487	< 0.001
	70 keV	4.29 ± 1.23	5.96 ± 1.88	4.874	< 0.001
	80 keV	4.39 ± 1.14	5.46 ± 1.69	3.457	0.001
	90 keV	4.07 ± 1.11	5.08 ± 1.41	3.632	< 0.001
	100 keV	3.91 ± 1.03	5.36 ± 1.57	3.190	0.002
	Conventional linear blending	4.14 ± 1.13	5.36 ± 1.57	4.104	< 0.001
CNR	40 keV	1.39 (0.47–2.13)*	3.81 (2.19–5.61)*	-5.689	< 0.001
	50 keV	0.87 (-0.29-1.50)*	2.95 (1.83–4.72)*	-5.662	< 0.001
	60 keV	0.44 (-0.12-1.16)*	2.25 (1.46–3.74)*	-6.065	< 0.001
	70 keV	0.23 (-0.36-0.81)	1.86 (1.03–3.23)*	-6.147	< 0.001
	80 keV	0.02 (-0.49-0.49)	1.04 (0.33–2.25)	-5.199	0.001
	90 keV	-0.11 (0.54 − 0.25)	0.56 (-0.14-1.31)*	-4.127	< 0.001
	100 keV	-0.19 (-0.68-0.27)*	0.38 (-0.15-0.95)*	-4.352	0.002
	Conventional linear blending	0.07 (-0.41-0.43)	0.96 (0.46–1.74)	-5.030	< 0.001

Note: Data were expressed as mean ± standard deviation (SD) or median (interquartile range, IQR). *P < 0.05 indicates a significant difference when compared to the Conventional linear blending group in CNR.

### Subjective evaluation of imaging quality

Table 4 presents the results of subjective image quality evaluations. In summary, compared with the conventional linear blending group, subjective scores for the 40 keV and 50 keV groups were significantly higher for both prostatic hyperplasia and PCa (*P* < 0.05), whereas scores for the 90 keV and 100 keV groups were significantly lower (*P* < 0.05). Additionally, subjective scores decreased progressively with increasing energy levels (40 keV, 50 keV, 60 keV, 70 keV, 80 keV, 90 keV, and 100 keV). The higher subjective scores for the 40 keV and 50 keV groups suggest improved image quality and lesion visibility in both prostatic hyperplasia and PCa, indicating potential clinical utility for enhancing diagnostic confidence and accuracy in DECT imaging.

**Table 4:** Comparison of subjective scores of the image quality in various monoenergetic reconstruction keV levels (40–100 keV) and conventional linear blending images

Groups	Prostatic hyperplasia (*n* = 46)	Prostate cancer (*n* = 37)	z	*P*
40 keV	5(4–5)*	5(4–5)*	-0.604	0.546
50 keV	5(4–5)*	5(4–5)*	-1.141	0.254
60 keV	4(3–5)	4(3–5)	-0.888	0.375
70 keV	4(3–4)	4(3–4)	-0.622	0.534
80 keV	3(2–4)	3(2–4)	-0.690	0.490
90 keV	3(2–3)*	3(2–3)*	-0.341	0.733
100 keV	2(1.5–2.5)*	2(1–3)*	0.475	0.635
Conventional linear blending	4(2.5-4)	3(3–4)	-0.562	0.574

Note: Data were expressed as median (interquartile range, IQR). *P < 0.05 vs. Conventional linear blending

### Diagnostic efficacy of DECT for PCa

**Fig. 2:**
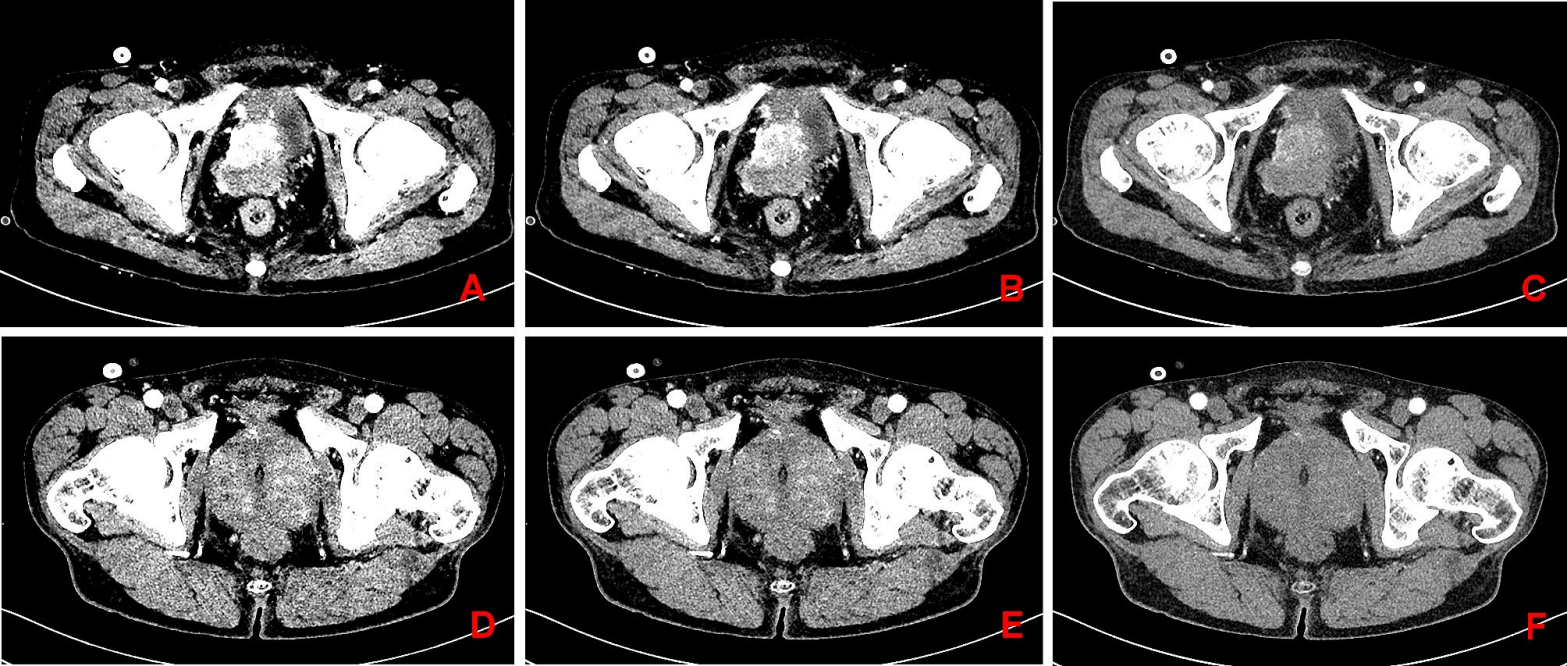
Comparison of CT values and CNR at 40 keV, 50 keV, and conventional linear blending images in patients with prostate cancer and prostate hyperplasia (Window Width: 300 / Window Level: 40). (**A**-**C**) The CT values of prostate cancer at 40 keV (**A**), 50 keV (**B**), and conventional linear blending image (**C**) were 246.8 HU, 175 HU, and 84.6 HU, respectively; CNRs were 6.75, 5.90, and 2.55, respectively. (**D**-**F**) The CT values of prostate hyperplasia at 40 keV (**D**), 50 keV (**E**), and conventional linear blending image (**F**) were 101.5 HU, 81.9 HU, and 54.5 HU; CNRs were 1.12, 0.75, and 0.11, respectively

Building upon the preceding results, both the 40 keV and 50 keV groups demonstrated significantly increased CT values and CNR values compared to conventional linear blending images (Fig. 2). Therefore, we conducted ROC curve analysis to compare the diagnostic efficacy of 40 keV, 50 keV, and conventional linear blending images for PCa.

**Fig. 3:**
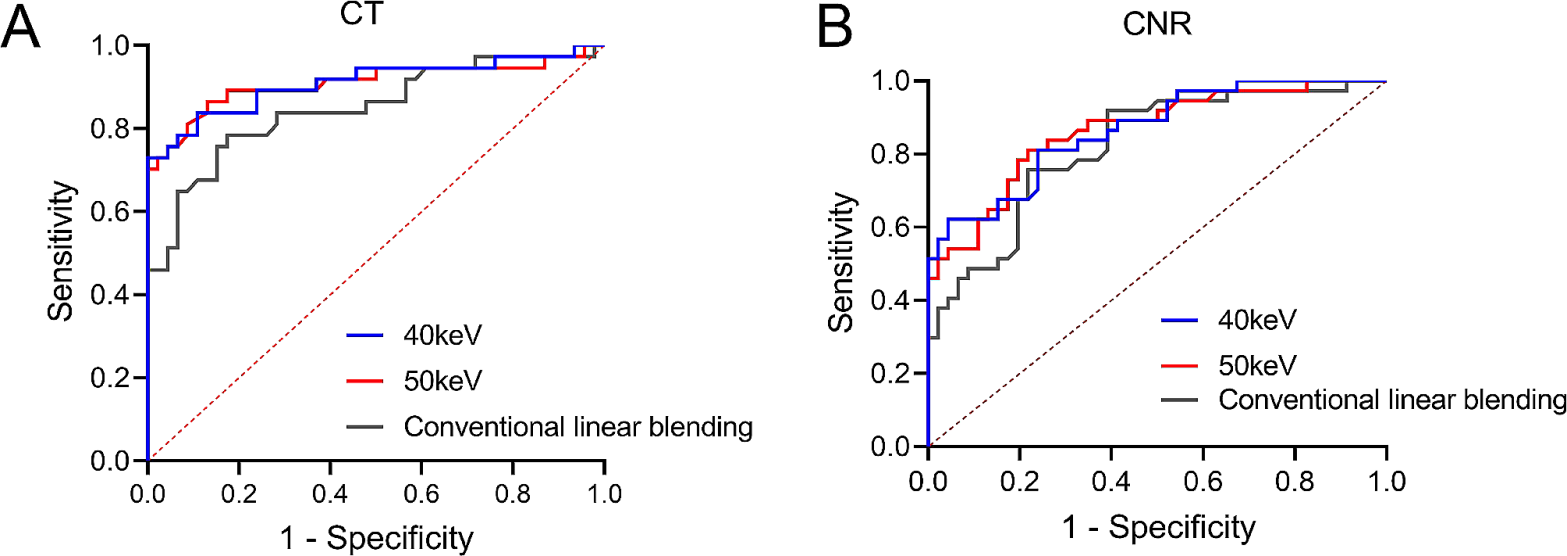
Receiver operating characteristic (ROC) curve for the prediction of prostate cancer using 40 keV, 50 keV, and conventional linear blending images. Subplots (**A**) and (**B**) display ROC curves based on computed tomography (CT) values and contrast-to-noise ratio (CNR) values, respectively

The results (Fig. 3; Table 5) revealed notable differences in the predictive performance of 40 keV, 50 keV, and conventional linear blending images based on their CT and CNR values, reflected in the respective areas under the receiver operating characteristic curve (AUC). Specifically, the AUC values for 40 keV, 50 keV, and conventional linear blending images in CT values were 0.910, 0.910, and 0.849, respectively. Correspondingly, the AUC values in CNR values were 0.865, 0.863, and 0.821, respectively. Importantly, both the 40 keV and 50 keV groups exhibited higher AUC values in CT than the conventional linear blending group, with CT values demonstrating superior diagnostic efficacy compared to CNR values. Thus, DECT at 40 keV and 50 keV could enhance PCa diagnostic accuracy with higher CT and CNR values and superior AUC in ROC analysis compared to conventional imaging, suggesting improved patient management.

**Table 5:** Analysis results of receiver operating characteristic curve

Variables	40 keV		50 keV	Conventional linear blending
CT values	AUC (95% CI)	0.910(0.839–0.982)	0.910 (0.835–0.984)	0.849 (0.761–0.936)
	*P* value	< 0.001	< 0.001	< 0.001
	Cut-off value	132.40	90.25	58.85
	Sensitivity, %	72.97	86.49	78.38
	Specificity, %	100%	86.96	82.61
CNR values	AUC (95% CI)	0.865(0.788–0.941)	0.863(0.784–0.941)	0.821(0.732–0.911)
	*P* value	< 0.001	< 0.001	< 0.001
	Cut-off value	2.92	1.58	0.52
	Sensitivity, %	62.16	81.08	75.68
	Specificity, %	95.65	78.26	78.26

Note: AUC, areas under the receiver operating characteristic curve; CI, confidence interval; CT, computed tomography; CNR, contrast-to-noise ratio

## Discussion

This study investigated the impact of virtual monoenergetic images reconstructed by DECT during the arterial phase on the image quality of prostate lesions and diagnostic performance for PCa, revealing significant advantages associated with low-energy virtual monoenergetic images, specifically at 40 keV and 50 keV, in subjective and objective evaluations and diagnostic efficiency. Overall, our findings support the enhanced detection capabilities of DECT, particularly in identifying csPCA and more advanced stages of PCa. Among the prostate cancer cases detected, 36 (97.3%) were classified as csPCA based on a Gleason score ≥ 7 and/or evidence of extraprostatic extension. Additionally, T stages varied, with the majority being T4 stages, indicating that the cancer had grown outside the prostate gland. These findings highlight the enhanced detection capabilities of DECT for csPCA and more advanced prostate cancers.

Our results indicate that the average CT attenuation values of prostate hyperplasia and PCa tumors in reconstructed images at 40–60 keV were significantly higher compared to those in the conventional linear blending group, suggesting enhanced contrast for these conditions at low-energy level and aligning with prior research that demonstrated low-energy virtual monoenergetic images can enhance tumor visualization and reduce the need for additional diagnostic scans [23]. This enhancement could be attributed to the predominance of the photoelectric effect in low-energy virtual monoenergetic images, which closely approximates the K-absorption edge of iodine, thereby increasing tissue contrast and improving soft tissue resolution [24]. Virtual monoenergetic images at 40 keV during the arterial phase demonstrate high clinical utility. They improve the detection sensitivity of PCa lesions compared to conventional CT scanning, potentially identifying invisible or challenging-to-detect cancers as well as the assessment of bone metastasis, lymph node involvement, and other organ conditions in a single examination session [25]. Thus, DECT holds promise as a novel imaging modality for PCa assessment and could serve as a complementary tool in cases where MRI is contraindicated [26, 27].

Furthermore, the CNR values of the 40–60 keV groups between patients with prostate hyperplasia or PCa were significantly higher than those of the conventional linear blending group, while the SNR values at each energy level were not significant compared to the conventional linear blending group. Higher SNR indicates clearer details and better overall image quality when the signal is much stronger than the noise [28], and a higher CNR signifies greater contrast and more distinct delineation between the target and surrounding tissues [29], essential for accurate diagnosis [30]. Our findings suggest that the observed enhanced image quality improved PCa detection rates, consistent with research by Geng et al., who demonstrated that DECT could enhance the identification and objective evaluation of laryngeal squamous cell carcinoma using low-energy virtual monoenergetic images [31].

In terms of subjective evaluation, our study found that the subjective scores of the 40 keV and 50 keV groups were significantly higher than those of the conventional linear blending group, suggesting that radiologists could more easily identify and evaluate prostate tumors on these low-energy images. This finding aligns with the findings of Wang et al., who highlighted that DECT enhances radiologists’ ability to detect breast cancer, thereby enhancing the clinical utility of DECT [24]. Regarding diagnostic efficacy for PCa, virtual monoenergetic images at 40 keV and 50 keV exhibited higher AUC values in CT values (0.91) compared to CNR values (0.86), and both were superior to conventional linear blending images. This suggests that in our study, virtual monoenergetic images at 40 keV and 50 keV may offer the best diagnostic performance for PCa. These results are consistent with research by Luo et al., who demonstrated that low-energy virtual monoenergetic images under DECT provide superior accuracy in characterizing tumors [32]. The application of virtual monoenergetic images at 40 keV and 50 keV could potentially aid clinical decision-making in future research and clinical practice.

In recent years, machine learning (ML) and deep learning (DL) have shown significant potential across biomedical fields, including medical imaging and drug discovery. These technologies can notably enhance DECT by optimizing image reconstruction, improving contrast differentiation, and reducing noise, particularly in the critical 40–60 keV energy range. For instance, ML models have advanced drug permeability studies in maternal-fetal medicine [33] and optimized drug delivery systems through blood-brain barrier re-routing [34]. DL techniques, such as age and gender estimation from electrocardiogram signals [35], illustrate their efficacy in processing complex biomedical data. Integrating ML and DL into DECT imaging could refine virtual monoenergetic parameters, enhancing lesion detectability and diagnostic confidence. Furthermore, leveraging DL’s abilities in managing extensive datasets could potentially improve DECT’s diagnostic precision by enabling more robust analysis and interpretation of imaging data [36]. These approaches could lead to enhanced clinical decision-making and patient outcomes in disease diagnosis. Moreover, our findings align with existing literature that highlights the potential of DL in enhancing DECT imaging. For instance, Cong et al. demonstrated that DL can generate high-quality VM images from single-spectrum CT images with high accuracy and low relative error [37], suggesting similar benefits in reducing system complexity and radiation dose compared to conventional DECT, which also corroborated with other related research studies [38, 39]. Additionally, Greffier et al. and Seo et al. emphasized the superior image quality and diagnostic performance of DL-enhanced DECT images in various clinical contexts [40, 41]. Herein, we demonstrated that VM images at 40 keV and 50 keV significantly improve CT attenuation values, CNR and diagnostic accuracy for PCa, corroborating the efficacy of DL-enhanced DECT imaging in clinical diagnostics and highlighting the potential of integrating DL techniques into DECT imaging to optimize cancer detection and characterization, ultimately improving clinical outcomes and reducing the need for invasive diagnostic procedures.

This study had several limitations to acknowledge. Firstly, as a single-center study with a relatively small sample size, the generalizability of our findings may be limited. Secondly, ongoing advancements in DECT scanning parameters and reconstruction algorithms necessitate further validation of our conclusions in future research. Thirdly, the absence of MRI data limits comprehensive comparative analysis of diagnostic performance. Additionally, our study predominantly included patients with advanced PCa and elevated PSA levels, limiting representation of earlier-stage cases, and the exclusion of lesions < 1 cm restricts DECT’s applicability in detecting smaller, early-stage PCa.

## Conclusion

In conclusion, DECT shows substantial potential to enhance the diagnostic capabilities for PCa through the reconstruction of virtual monoenergetic images at low-energy levels (40 keV and 50 keV) during the arterial phase. This innovative technology not only enhances image quality but also improves diagnostic accuracy, thereby playing a crucial role in enhancing early detection and facilitating tailored treatment planning for PCa. Future research will aim to further validate and expand upon our findings, aiming to optimize the clinical application of DECT technologies and deliver more precise diagnoses and personalized treatment strategies to patients.

## Data Availability

The datasets used and/or analyzed during the current study are available from the corresponding author on reasonable request.

## Abbreviations

BMI: Body mass index
CNR: Contrast-to-noise ratio
CT: Computed tomography
DECT: Dual-energy computed tomography
FPSA: Free PSA
IQR: Interquartile range
MRI: Magnetic resonance imaging
PSA: Prostate-specific antigen
ROC: Receiver operating characteristic
SD: Standard deviation
SNR: Signal-to-noise ratio
TPSA: Total PSA
